# Cerebral ischemia induces the aggregation of proteins linked to neurodegenerative diseases

**DOI:** 10.1038/s41598-018-21063-z

**Published:** 2018-02-09

**Authors:** Anja Kahl, Ismary Blanco, Katherine Jackman, Juhi Baskar, Harihar Milaganur Mohan, Reunet Rodney-Sandy, Sheng Zhang, Costantino Iadecola, Karin Hochrainer

**Affiliations:** 1 Feil Family Brain and Mind Research Institute, Weill Cornell Medicine, New York, NY10065 USA; 2 Institute of Biotechnology and Life Sciences Biotechnologies, Cornell University, Ithaca, NY14853 USA

## Abstract

Protein aggregation critically affects cell viability in neurodegenerative diseases, but whether this also occurs in ischemic brain injury remains elusive. Prior studies report the post-ischemic aggregation of ubiquitin, small ubiquitin-related modifier (SUMO) and ribosomes, however whether other proteins are also affected is unknown. Here we employed a proteomic approach to identify the insoluble, aggregated proteome after cerebral ischemia. Mice underwent transient middle cerebral artery occlusion or sham-surgery. After 1-hour reperfusion, prior to apparent brain injury, mice were sacrificed and detergent-insoluble proteins were obtained and identified by nanoLC-MS/MS. Naturally existing insoluble proteins were determined in sham controls and aggregated proteins after cerebral ischemia/reperfusion were identified. Selected aggregated proteins found by proteomics were biochemically verified and aggregation propensities were studied during ischemia with or without reperfusion. We found that ischemia/reperfusion induces the aggregation of RNA-binding and heat-shock proteins, ubiquitin, SUMO and other proteins involved in cell signalling. RNA-binding proteins constitute the largest group of aggregating proteins in ischemia. These include TDP43, FUS, hnRNPA1, PSF/SFPQ and p54/NONO, all of which have been linked to neurodegeneration associated with amyotrophic lateral sclerosis and frontotemporal dementia. The aggregation of neurodegeneration-related disease proteins in cerebral ischemia unveils a previously unappreciated molecular overlap between neurodegenerative diseases and ischemic stroke.

## Introduction

Protein aggregation is a pathological hallmark of neurodegenerative diseases (NDs), such as Alzheimer’s disease (AD), Parkinson’s disease (PD), Huntington’s disease (HD), amyotrophic lateral sclerosis (ALS) and frontotemporal dementia (FTD). In these diseases the aggregation of different proteins has been implicated in distinctive disease pathogeneses. For example, in AD extracellular amyloid-beta (Aβ) accumulation is linked to excitotoxicity causing neuronal dysfunction and death^1,2^. In PD, the aggregation of α-synuclein is thought to interfere with synaptic transmission, resulting in synaptic failure^3,4^, whereas in HD, huntingtin aggregation is involved in the transcriptional repression of BDNF and PGC-1α genes, mitochondrial dysfunction and defective axonal transport^5–7^. Finally, aggregation of RNA-binding proteins (RNABPs) TDP43 and FUS has been linked to ALS and FTD, where it may alter RNA processing^8–10^. Taken together, these findings suggest that the formation of protein aggregates differentially affects distinct cellular processes in NDs, which may lead to neuronal dysfunction and death.

There is increasing evidence that protein aggregation also occurs in the acute neuronal injury induced by cerebral ischemia^11–16^. However, the identity of these aggregated proteins and their relationship to proteins accumulating in ND has not been established. In this study we employed a mouse model of middle cerebral artery occlusion (MCAO) combined with nano liquid chromatography (LC)-mass spectrometry (MS)/MS to identify proteins that aggregate during cerebral ischemia/reperfusion (I/R) injury. Our results reveal an unexpected similarity between protein aggregates in chronic neurodegeneration and acute ischemic injury, which may suggest a formerly unrecognized link between the molecular pathology of neurodegenerative diseases and stroke.

## Results

### Ischemic stroke induces protein aggregation

**Figure 1:**
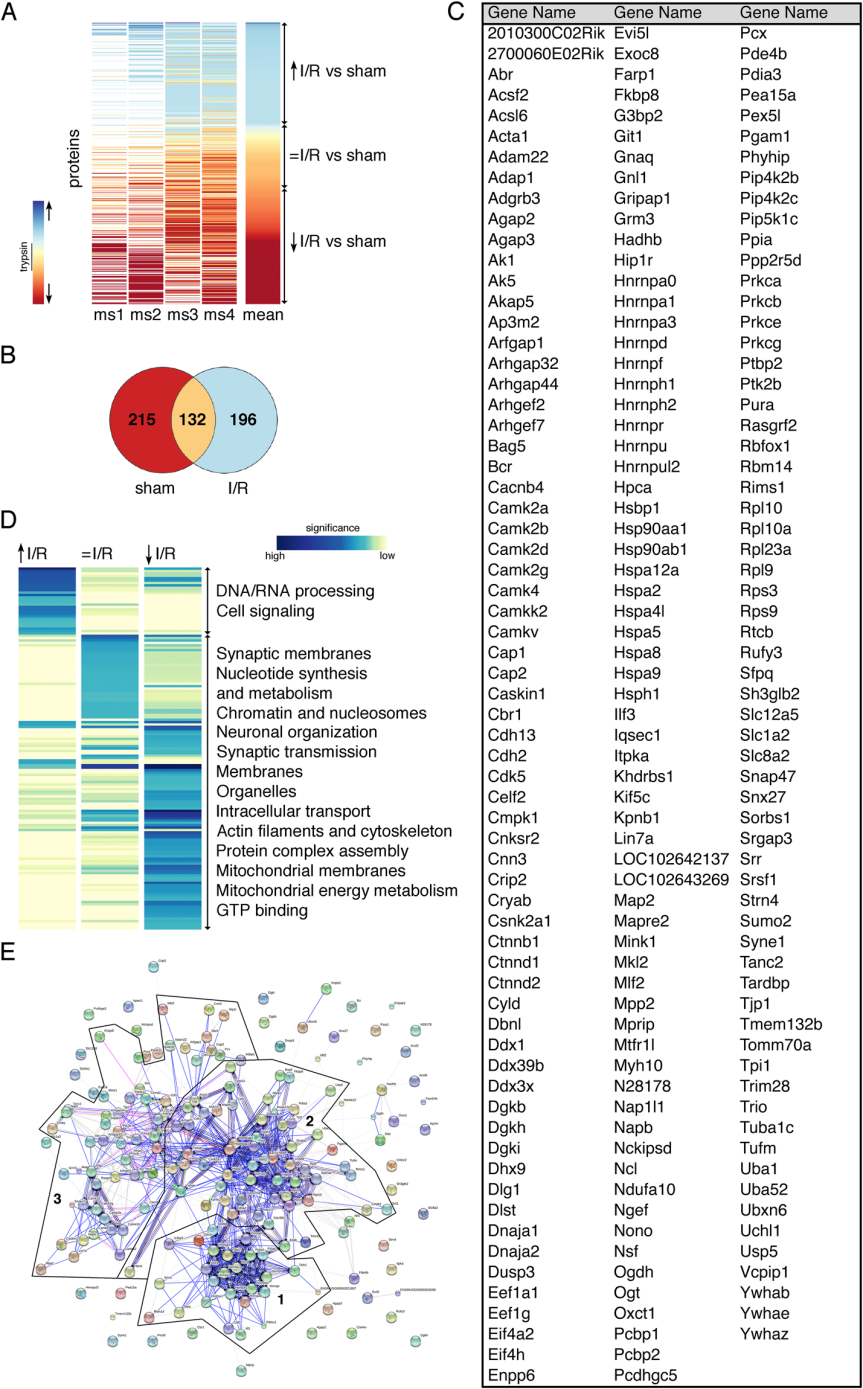
Ischemic stroke leads to the aggregation of 196 highly interconnected proteins that are mainly associated with RNA processing, stress response and cell signaling. (**A**) Ipsilateral neocortical Triton-insoluble proteins were identified after sham-surgery or MCAO and 1-hour reperfusion (I/R). The heat map depicts relative levels of Triton-insoluble proteins found in I/R compared to sham-controls. Each horizontal line represents a single protein, the vertical strips depict four independent mass spectrometry runs (ms1-4) and the mean value of the 4 runs. Trypsin levels served as internal standard for the calculation of fold changes of protein levels in I/R versus sham conditions. Proteins were clustered into three groups dependent on their solubility before and after I/R (increased, ↑, blue shaded), (unchanged, = , yellow/light orange shaded), (reduced, ↓, dark orange/red shaded). Upward and downward pointing arrows indicate an increase or decrease in relative I/R versus sham insoluble protein levels, respectively. ms, mass-spectrometry. (**B**) A Venn diagram showing the distribution of Triton-insoluble proteins in sham conditions and after I/R is shown. (**C**) An alphabetical list of the 196 aggregated proteins (↑) is provided. (**D**) Proteins from each induction group identified in Fig. 1A were examined for GO term enrichment through the DAVID database. Similar enrichment terms were clustered and the natural logarithm of corrected P values was plotted. Only terms with an enrichment score of P < 10^–5^ in at least one group were considered for analysis. Blue and yellow shades represent high and low significance, respectively. (**E**) Aggregated proteins in I/R (↑) were analyzed for functional association networks via the STRING database. Strong association networks were compiled into three main clusters (clusters 1, 2, 3). A high-resolution image of Fig. 1E is included as Supplementary Figure 1.

To identify aggregated proteins during I/R we subjected mice to MCAO or sham-surgery and harvested ipsilateral neocortical tissue after 1-hour reperfusion. This time-point was chosen based on the prominent cortical aggregation of protein-modifiers ubiquitin and SUMO2/3 we had previously detected^12,13^. Proteins resistant to 2% Triton X100 solubilization were isolated, subjected to trypsin digestion and analysed by nanoLC-MS/MS followed by label free quantitation (Supplementary Tables S9 to S11). We first determined inherently Triton-insoluble proteins in sham animals. Then, we identified newly appearing insoluble proteins, which we define as aggregates, in animals that underwent I/R injury. In total, 543 high confidence proteins were identified in both sham and ischemia-derived Triton-insoluble fractions across four independent mass spectrometry runs. While 132 proteins remained unchanged, the solubility of 411 proteins was altered between sham and ischemic conditions (Fig. 1A,B). Out of these, 196 proteins showed increased Triton-insolubility during I/R, indicating aggregation (Fig. 1A–C). Some of the detected proteins, such as ubiquitin (Uba52) and SUMO, as well as chaperones Hsc70, Hsp40, Hsp70 and translation initiation factor eIF4 were previously reported to aggregate after ischemic stroke^12–16^, confirming the validity of our approach. Therefore, I/R leads to appreciable protein aggregation, which may critically influence post-ischemic cell fate.

### Ischemic stroke leads to aggregation of proteins with roles in DNA/RNA processing, stress response and cell signalling

To predict the functional consequences of protein aggregation after ischemic stroke we assessed the Gene Ontology (GO) term enrichment in all groups from Fig. 1A. While proteins with high insolubility under sham conditions were largely associated with generally known Triton-insoluble cellular material, such as synaptic membranes, histones, mitochondria and cytoskeleton, proteins with increased insolubility during I/R were related to protein classes that are normally not associated with Triton-insolubility, such as DNA/RNA processing and signal transduction proteins (Fig. 1D, Supplementary Tables S12 and S13). Hence, these proteins shifted from a Triton-soluble to a Triton-insoluble state after I/R, indicating formation of Triton-inaccessible, insoluble aggregates. To further examine whether aggregating proteins during I/R are functionally connected, we performed STRING database analysis for functional association networks. We found that aggregating proteins form two dense clusters that are highly interconnected, indicating a strong association (Fig. 1E, clusters 1 + 2). Proteins within these clusters were linked to RNA processing (e.g. Hnrnpa0, Hnrnpa1, Pcbp1, Nono, Tardbp, Sfpq, Eif4a, Eif4h), co-clustering with stress response proteins (e.g. Hspa5, Dnaja2, Hspa2, Hspa9). We also detected a looser cluster largely consisting of cell signaling molecules (e.g PKC-α, -β, -γ, -ε, CaMKII-α, -β, -γ, -δ, Arhgap-32, −44) (Fig. 1E, cluster 3), substantiating earlier results. These data suggest that due to aggregation of involved proteins, pathways related to DNA/RNA processing, stress response and signal transduction are altered after ischemic stroke.

### Ischemic stroke induces the aggregation of ALS- and FTD-related RNABPs

**Figure 2:**
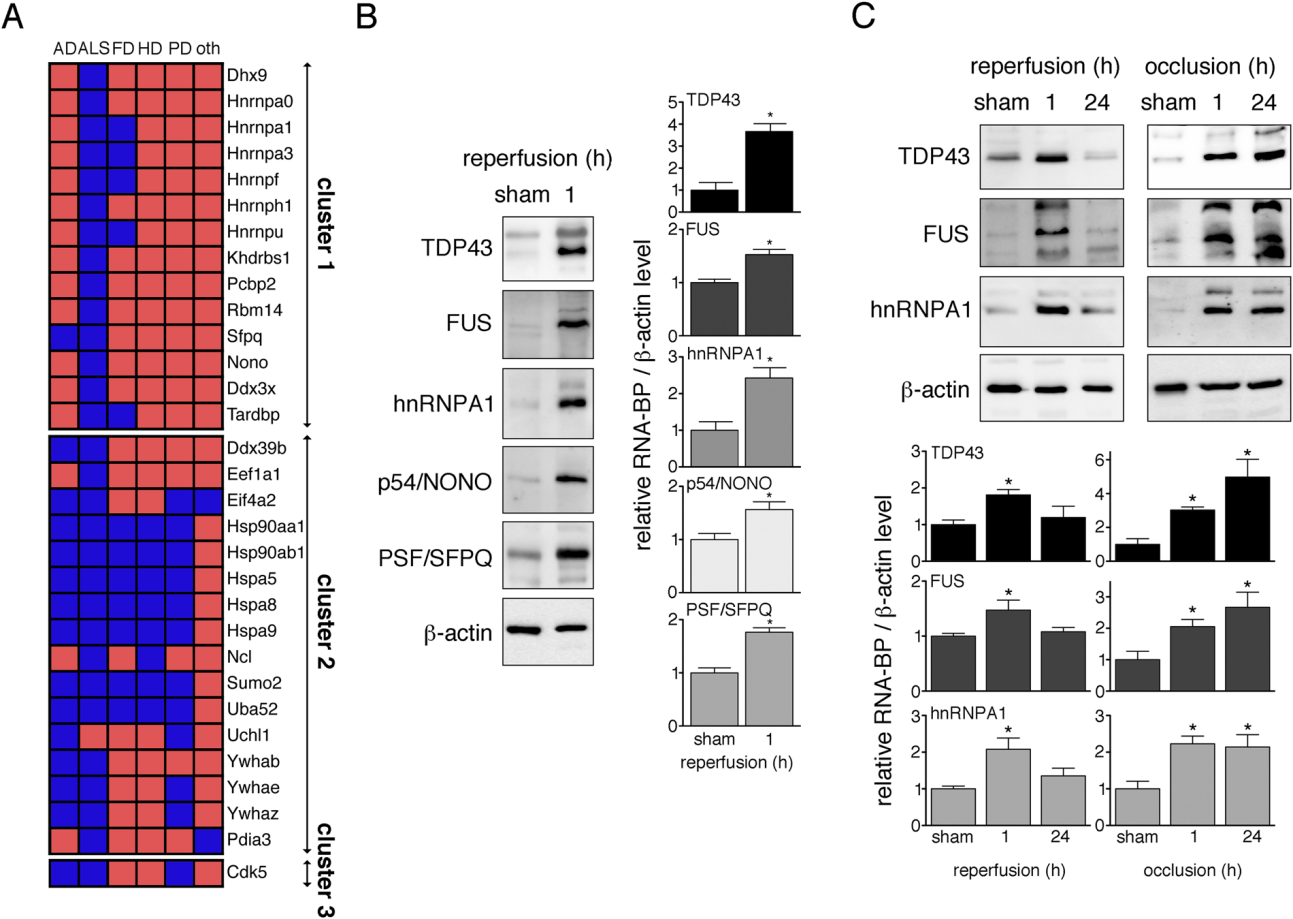
Ischemic stroke induces the aggregation of the ALS- and FTD-related RNABPs TDP43, FUS, hnRNPA1, PSF/SFPQ and p54/NONO. (**A**) I/R-derived aggregated proteins were analyzed for their known ability to form aggregates in neurodegeneration. Identified proteins were clustered according to analysis in Fig. 1D. The heatmap points out disease affiliation of aggregate-prone proteins in ND, where blue boxes indicate positive and red boxes negative hits. AD, Alzheimer’s disease; ALS, amyotrophic lateral sclerosis; FD, frontotemporal dementia; HD, Huntington’s disease; oth, other; PD, Parkinson’s disease. (**B**) Triton-insoluble fractions were obtained from ipsilateral neocortical tissue from mice subjected to sham surgery or MCAO followed by 1-hour reperfusion. The presence of the RNABPs TDP43, FUS, hnRNPA1, p54/NONO and PSF/SFPQ in Triton-insoluble fractions was detected by Western Blotting with respective antibodies. Optical densities of RNABP bands were measured and normalized to β-actin. Changes in RNABPs were expressed relative to sham controls. *P < 0.05 from sham; n = 9/group. (**C**) Ipsilateral neocortices from sham animals, after MCAO followed by 1 and 24 hours reperfusion, or after 1 and 24 hours permanent MCAO were harvested. Triton-insoluble proteins were isolated and examined for the presence of TDP43, FUS and hnRNPA1 by Western Blotting. Quantification of results was carried out as in Fig. 2B. Full-size blots are presented in Supplementary Figures S2 and S3. *P < 0.05 from sham; n = 3–6/group. h, hours.

Screening the literature we found that out of the 196 identified aggregating proteins during I/R, 31 were previously published in connection with neurodegeneration-associated protein aggregation (Supplementary Table S14). When clustered into the functional networks identified by STRING in Fig. 1D, only one out of 54 proteins in the cell signalling cluster aggregated during ND (cluster 3, Fig. 2A). However, in clusters 1 + 2 containing RNA-binding and stress response proteins, 30 of the 85 proteins aggregated in ND (Fig. 2A). Notably, 15 aggregating proteins after ischemic stroke were RNABPs linked to ALS and FTD, among them TDP43 and hnRNPA1^8,17^, as well as PSF/SFPQ and p54/NONO, two paraspeckle proteins with emerging roles in ALS^18,19^. In addition, in one of the four proteomics analyses we found evidence of aggregation of FUS, another well-described protein linked to ALS^10^.

To provide biochemical confirmation of aggregation of these RNABPs, we analysed Triton-insoluble neocortical homogenates from sham and ischemic mice by Western Blot. All tested proteins were only marginally detected in Triton-insoluble fractions in the sham group, but were significantly aggregated in ischemic mice (Fig. 2B), thus confirming nanoLC-MS/MS results.

We previously reported that ubiquitin aggregation after ischemia is reperfusion-dependent^12^. Here we sought to determine whether this is also the case for RNABPs. To this end we examined TDP43, FUS and hnRNPA1 presence in Triton-insoluble fractions after MCAO/reperfusion, as well as after permanent MCAO without reperfusion. As before, all three proteins were detected in aggregates after 1-hour reperfusion (Fig. 2C, left panel). However, TDP43, FUS and hnRNPA1 also aggregated with permanent ischemia (Fig. 2C, right panel), suggesting that unlike ubiquitin^12^ RNABPs accumulate during the ischemic period independently of reperfusion.

Ubiquitin aggregation induced in early reperfusion is transient^12^. To assess the reversibility of ischemic RNABP aggregation, we tested their aggregation levels in both MCAO/24-hour reperfusion and 24-hour permanent MCAO. After 24 hours of reperfusion no aggregated TDP43, FUS or hnRNPA1 was observed (Fig. 2C, left panel), indicating that like ubiquitin RNABPs accumulate reversibly in presence of reperfusion. In contrast, at 24-hour permanent MCAO we found persistent aggregation of all RNABPs (Fig. 2C, right panel), suggesting that reperfusion is required for the resolution of these aggregates. Therefore, ischemia-induced aggregation of RNABPs is reversible by reperfusion.

## Discussion

We show here that protein aggregation that is commonly linked to chronic NDs is also prevalent in acute ischemic brain injury. The majority of proteins that aggregate during I/R are related to RNA processing, stress response and signal transduction, processes previously reported to be critically affected by I/R. Alteration of heat shock proteins^20^ as well as RNA-to-protein processing^21^ are integral parts of the ischemic stress response. Also, the activity of cell signalling molecules, such as PKC^22,23^, CaMKII^24,25^, Uchl1^26,27^ and 14-3-3^28,29^, all of which we found aggregated, is modified by ischemia. We performed our experiments at 1-hour reperfusion before any evidence of cell death is observed in either striatal or cortical tissue^30^. Therefore it is unlikely that aggregation is the consequence of cell death. Rather, we hypothesize that aggregation alters protein function to modulate impeding acute tissue injury. Some of the detected proteins have been shown to be protective in ischemic models, such as Hsp70^31–33^, SUMO^34,35^ and 14-3-3^36^, while others are detrimental, e.g. Hsp90^33,37^ and CaMKII^24^, suggesting a diversity of functions and roles.

Our study indicates a predominant aggregation of RNA-binding and heat-shock proteins, ubiquitin, SUMO, and other signaling molecules during I/R. The mechanism by which this aggregation occurs after ischemia remains to be established. However there are several possibilities to be considered. First, RNABPs commonly contain prion-like domains that are essential for their physiological functions, but make them inherently aggregation-prone. Prion-like domains are required for formation of subnuclear membrane-less structures, such as hnRNP complexes and paraspeckles^38^, and critically regulate stress responses by enabling stress granule formation, as seen in case of TDP43 and FUS^39,40^. Hence the aggregation of RNABPs after ischemia may be induced by post-ischemic stress. The detection of ubiquitin and SUMO in aggregates on the other hand may be due to preferential modification of aggregation-prone proteins, such as RNABPs, however this is purely speculative at this stage. Finally, as previously suggested, post-ischemic protein aggregation may result from accumulation of misfolded proteins that cannot be refolded and are not properly targeted for proteasomal degradation^14,41,42^. This could explain the presence of ubiquitin and heat-shock proteins in post-ischemic aggregates.

The largest number of I/R-derived aggregated proteins is linked to RNA processing. Whereas certain cytosolic proteins involved in protein synthesis, like eukaryotic translation factors and ribosomal subunits are known to become insoluble post-ischemia^16^, aggregation of nuclear RNA processing proteins has not been previously described. The sequestration of nuclear RNABPs in aggregates may be the cause or consequence of the well-described translational shutdown that accompanies ischemic stroke^16,43–45^. We find a large number of hnRNP proteins (hnRNPA0, hnRNPA1, hnRNPA3, hnRNPD, hnRNPE1 (PCBP1), hnRNPE2 (PCBP2), hnRNPF, hnRNPH1, hnRNPH2, FUS (hnRNPP2), hnRNPR, hnRNPU) as well as essential components of paraspeckles (NONO, PSF, Rbm14) aggregated during I/R, which implies that their structure is substantially changed. The consequences on associated functions in nucleocytoplasmic RNA transport and RNA processing remain to be investigated.

A key finding of the present study is that ischemia induces aggregation of RNABPs with a strong link to ALS/FTD. A hallmark of these diseases is the irreversible aggregation of TDP43 and FUS^8,10^. In addition to these two proteins, other RNABPs either co-aggregate (PSF and NONO)^46–48^ or may also directly aggregate through mutations (hnRNPA1)^17^. Our data show that, at variance with ALS/FTD, post-ischemic RNABP aggregation is reversible upon reperfusion. Such reversible aggregation is common to stress responses requiring stress granule formation, which has also been described in ischemic stroke^45,49^. In contrast to I/R and similar to ALS/FTD, we found that RNABP aggregation is irreversible in ischemia without reperfusion. The difference between the two injury models may offer a great opportunity to study RNABP aggregation and disaggregation mechanisms.

In summary, our study provides the first comprehensive proteomic analysis of post-ischemic aggregated proteins. Unexpectedly, we found that nuclear RNABPs implicated in ALS/FTD also aggregate in acute cerebral ischemia. Although the pathogenic implications of RNABP aggregation in ischemic brain injury remains to be defined, the data suggest a parallelism in protein aggregation between mechanistically distinct diseases and unveil a potential new mechanism of cell dysfunction and death following brain ischemia.

## Materials and Methods

### MCAO ischemia model

All animal procedures were approved by the Institutional Animal Care and Use Committee (IACUC) of Weill Cornell Medicine and were carried out according to IACUC, NIH, and ARRIVE guidelines (http://www.nc3rs.org/ARRIVE). Experiments were performed in 7–9 weeks old male C57Bl/6 J mice (Jackson Laboratory, Bar Harbor, ME, USA) with an average body weight of 22.2 ± 1.9 grams. Cerebral I/R was induced using the intraluminal filament method as previously described^12,50^. Briefly, mice were anesthetized with isoflurane (1.5–2.0%) and the middle cerebral artery (MCA) was occluded using a heat-blunted 6–0 nylon suture, which was introduced into the right external carotid artery and then progressed along the internal carotid artery. At the same time, the right common carotid artery was ligated. The filament stayed in place for the duration of 35 minutes (ischemia), after which reperfusion was initiated by withdrawal of the suture. The filament was not retracted in animals undergoing permanent ischemia. Cerebral blood flow (CBF) was monitored with transcranial laser-Doppler flowmetry (Periflux System 5010, Perimed, PA, USA) in the centre of the ischemic territory (2 mm posterior, 5 mm lateral to bregma). Only animals that exhibited a reduction in CBF of >85% during MCAO and in which CBF recovered by >80% after 10 min of reperfusion were included in this study. For sham surgery, vessels were visualized and cleared of connective tissue without performing any other manipulations. In all mice, rectal temperature was maintained at 37.0 °C ± 0.5 °C during the surgical procedure and in the recovery period using a heating pad (TC-1000, CWE Inc., Fort Wayne, IN, USA) until the animals regained full consciousness.

### Isolation of neocortical Triton-insoluble proteins

Triton-insoluble proteins were isolated from cortical tissue as described in detail in our previous publication^12^. Briefly, 2-mm cortical slices from the MCA territory (approx. +1.2 to −0.8 mm bregma) were mechanically homogenized in buffer containing 15 mM Tris pH 7.6, 250 mM sucrose, 1 mM MgCl_2_, 2.5 mM EDTA, 1 mM EGTA, 1 mM sodium orthovanadate, 5 mM sodium fluoride, 20 mM phenylphosphate, 20 mM N-ethylmaleimide, 1 mM dithiothreitol (DTT), 1x protease inhibitor cocktail (Roche Applied Sciences, Indianapolis, IN, USA). Pellets obtained by high-speed centrifugation were further sonicated in homogenization buffer (10 seconds at 20% amplitude) and then incubated for 1 hour in 2% Triton X100/150 mM KCl. Triton-insoluble proteins, defined by their resistance to Triton/KCl solubilization, were finally obtained by centrifugation at 13,000 rpm for 15 minutes. Triton-insoluble fractions derived from sham and ischemic animals were screened for the absence and presence of high molecular weight ubiquitin by Western Blotting with anti-ubiquitin antibody (ubi-1; Thermo Fisher Scientific, Waltham, MA, USA) to ensure experimental accuracy^12^.

### In-solution trypsin digestion and MS-associated sample preparation

In-solution digestion was performed following a published protocol^51^ with slight modifications. Triton-insoluble protein pellets (300–400 µg proteins) were washed twice with ice-cold acetone, air-dried, and dissolved by incubation and sonication in denaturing solution containing 8 M guanidine-HCl and 50 mM Tris pH8. After clearance through high-speed centrifugation, the denatured samples were reduced by addition of 90 mM Tris(2-carboxyethyl) phosphine at 60 °C for 45 minutes, and alkylated with 140 mM iodoacetamide for 1 hour at room temperature. Excess iodoacetamide was quenched with 15 mM DTT and samples were diluted with 100 mM Tris pH8 to reduce the guanidine-HCl concentration to less than 1 M. The samples (final volume of 650 µl) were digested by incubation with 400ng/µl trypsin (Promega, Madison, WI, USA) at 37 °C for 16 hours. Digestions were stopped by the addition of 10% TFA. The digests were further desalted by solid phase extraction using Sep-Pack Cartridges (Waters Corporation, Milford, MA, USA) and eluted peptides were evaporated to dryness using a Speedvac SC110 (Thermo Savant, Milford, MA, USA).

### NanoLC-MS/MS

Forty µg peptides were reconstituted in 4 µl 0.5% formic acid (FA) for nanoLC-MS/MS analysis, which was carried out by an Orbitrap Elite mass spectrometer (Thermo Fisher Scientific) equipped with a “CorConneX” nano ion source device (CorSolutions LLC, Ithaca, NY, USA). The Orbitrap was interfaced with a Dionex UltiMate3000RSLCnano system (Thermo Fisher Scientific). Peptides were injected onto a PepMap C18 trap column-nano Viper (5 µm, 100 µm x 2 cm, Thermo Fisher Scientific) at 20 μL/min flow rate for on-line desalting and then separated on a PepMap C18 RP nano column (3 µm, 75 µm x 25 cm, Thermo Fisher Scientific), which was installed in the nano device with a 10 µm spray emitter (NewObjective, Woburn, MA, USA). The Orbitrap calibration and nanoLC-MS/MS operation were executed as previously described^52^. Peptides were eluted with a 90-minute gradient of 5–38% acetonitrile (ACN) in 0.1% FA at a flow rate of 300nL/minute, followed by a 7-minute ramping to 95% ACN-0.1% FA and a 8-minute hold at 95% ACN-0.1% FA. The Orbitrap Elite was operated in positive ion mode with nano spray voltage set at 1.6 kV and source temperature at 250 °C. The instrument was operated in parallel data-dependent acquisition (DDA) under FT-IT mode using FT mass analyser for one MS survey scan from m/z 375 to 1800 with a resolving power of 120,000 (fwhm at m/z 400), followed by MS/MS scans of the top 15 most intensive peaks with multiple charged ions above a threshold ion count of 10,000 in FT mass analyser. External calibration using Ultramark 1621 for both FT mass analyser and IT mass analyser was performed. Dynamic exclusion parameters and normalized collisional energy were set as previously^52,53^. All data were acquired under Xcalibur 2.2 operation software (Thermo Fisher Scientific).

### Protein identification, label-free quantitation and data analysis

All MS and MS/MS raw spectra were generated as MGF files by Proteome Discoverer 1.4 (Thermo Fisher Scientific) for subsequent Mascot 2.3.02 search (Matrix Science, Boston, MA, USA) against the mouse RefSeq database (35,359 entries, downloaded on 7/25/2007 from NCBInr). Searches were performed with two-missed cleavage sites by trypsin allowed. The peptide and MS/MS tolerance were set to 10 ppm and 0.5 Da, respectively. A fixed carbamidomethyl modification of cysteine was set along with methionine oxidation, lysine ubiquitination and deamidation on asparagines/glutamine residues as variable modifications. Data filtering parameters were set to a peptide identity probability of 95% CI with peptide ion score cut-off at 30. All MS/MS spectra from initial database searches were re-inspected and validated using Xcalibur 2.2.

For relative quantitative analysis of peptides across samples, all raw spectra were searched using MaxQuant version 1.5.1.2 against the above mouse RefSeq database with the same setting on peptide/MS/MS tolerance and modifications. The estimated false discovery rate thresholds were specified at maximum 1%. Minimum peptide length was set to 6, and unique and razor peptide intensities were used. All other parameters in MaxQuant were set to default values^54^. Relative quantitation of identified proteins and peptides was determined by Label Free Quantitation (LFQ) intensities (the output of the MaxLFQ algorithm), which uses delaying normalization after summing up total peptide ion intensities for determining normalization factors via a global optimization procedure^55^.

The original data and a complete list of identified proteins can be found in Supplementary Tables S1 to S11. Relative protein levels I/R versus sham were calculated for each of the four mass spectrometry runs, and mean values were determined (8 mice/group). A total of 543 high confidence proteins were considered for analysis after removal of low confidence hits that were either only called in one nanoLC-MS/MS run or showed cross induction variations. Proteins were subdivided into three groups according to their induction level I/R versus sham. Trypsin that was added at equal concentrations to all samples was used as an internal standard to determine induction/reduction levels (1.04 ± 0.16) (Supplementary Table S8). Proteins with an induction higher than the trypsin+ range were marked as increased (↑), proteins lower than the trypsin - range were considered reduced in the Triton-insoluble fraction after stroke (↓). Proteins with induction/reduction levels within the trypsin range were classified as unchanged (=).

### Database searches

To assess the occurrence frequency of Gene Ontology (GO) terms in groups we used the Database for Annotation, Visualization and Integrated Discovery (DAVID) version 6.7 (https://david-d.ncifcrf.gov/). Functional annotation was performed for GO terms cellular component, molecular function and biological process (Supplementary Tables S12 and S13). A functional annotation chart was created setting the thresholds to Count 2 and EASE 0.1. Only annotation terms that had an enrichment score of Benjamini-corrected P < 10^–5^ in at least one group were considered for analysis. Functional protein associations within induction groups were determined through the Search Tool for the Retrieval of Interacting Genes/Proteins (STRING) database (https://string-db.org/) with a minimum required interaction score of 0.400.

Heat maps representing the data were generated in RStudio (RStudio Team (2015). RStudio: Integrated Development for R. RStudio, Inc., Boston, MA, http://www.rstudio.com/) with the heatmap.2 function using ‘gplots’ and ‘RColorBrewer’ packages.

### Western blotting and associated statistical analysis

For each sample, an equal amount of total proteins was loaded and resolved on 10% sodium dodecyl sulphate-polyacrylamide gel electrophoresis. Proteins were transferred to Immobilon-P membranes (EMD Millipore, Billerica, MA, USA), which were subsequently blocked in 5% non-fat milk/1x Tris Buffered Saline (TBS)-Tween20 and incubated overnight at 4 °C with primary antibodies (1:1000 dilution in 0.3% Bovine Serum Albumin/1x TBS-Tween20 (TDP43; rabbit IgG (10782-2-AP; ProteinTech, Rosemont, IL, USA), FUS/TLS; rabbit IgG (11570-1-AP; ProteinTech), hnRNPA1; mouse IgG_2b_ (clone 9H10; Thermo Fisher Scientific), p54/NONO; mouse IgG_2b_ (clone A11; Santa Cruz Biotechnology, Dallas, TX, USA), PSF/SFPQ; rabbit IgG (A301-321A; Bethyl Laboratories, Montgomery, TX, USA), β-actin; mouse IgG_1_ (clone AC-15; Sigma Aldrich, St. Louis, MO, USA)). Membranes were subsequently washed with 1x TBS/Tween20 and incubated with horseradish-peroxidase (HRP)-conjugated goat anti-mouse or anti-rabbit IgG (1:5000) (AffinitiPure; Jackson ImmunoResearch Laboratories, West Grove, PA, USA) secondary antibodies for 1 hour at room temperature. Western blots were developed using Clarity Western ECL substrate and a ChemiDoc Imaging System (Bio-Rad Laboratories, Hercules, CA, USA).

GraphPad Prism software (version 7.0, GraphPad Software, San Diego, CA, USA) was used for statistical analysis. Data are expressed as mean ± SEM. Statistical significance between multiple groups was assessed by one-way ANOVA followed by Bonferroni *post hoc* test. Two group comparisons were analysed by the two-tailed Student’s *t*-test. P values < 0.05 were considered statistically significant.

### Data availability

All data generated or analysed during this study are included in this published article and its Supplementary Information files.

## Electronic supplementary material

Supplementary Figures S1-S3

Supplementary Tables S1-S14

## References

[1] Masters CL (1985). Amyloid plaque core protein in Alzheimer disease and Down syndrome. Proc Natl Acad Sci USA.

[2] Mattson MP (1992). Beta-Amyloid peptides destabilize calcium homeostasis and render human cortical neurons vulnerable to excitotoxicity. J Neurosci.

[3] Garcia-Reitbock P (2010). SNARE protein redistribution and synaptic failure in a transgenic mouse model of Parkinson’s disease. Brain.

[4] Spillantini MG (1997). Alpha-synuclein in Lewy bodies. Nature.

[5] Cui L (2006). Transcriptional repression of PGC-1alpha by mutant huntingtin leads to mitochondrial dysfunction and neurodegeneration. Cell.

[6] Gauthier LR (2004). Huntingtin controls neurotrophic support and survival of neurons by enhancing BDNF vesicular transport along microtubules. Cell.

[7] Zuccato C (2001). Loss of huntingtin-mediated BDNF gene transcription in Huntington’s disease. Science.

[8] Neumann M (2006). Ubiquitinated TDP-43 in frontotemporal lobar degeneration and amyotrophic lateral sclerosis. Science.

[9] Neumann M (2009). A new subtype of frontotemporal lobar degeneration with FUS pathology. Brain.

[10] Vance C (2009). Mutations in FUS, an RNA processing protein, cause familial amyotrophic lateral sclerosis type 6. Science.

[11] Hayashi T, Takada K, Matsuda M (1992). Post-transient ischemia increase in ubiquitin conjugates in the early reperfusion. Neuroreport.

[12] Hochrainer K, Jackman K, Anrather J, Iadecola C (2012). Reperfusion rather than ischemia drives the formation of ubiquitin aggregates after middle cerebral artery occlusion. Stroke.

[13] Hochrainer K, Jackman K, Benakis C, Anrather J, Iadecola C (2015). SUMO2/3 is associated with ubiquitinated protein aggregates in the mouse neocortex after middle cerebral artery occlusion. J Cereb Blood Flow Metab.

[14] Hu BR (2001). Protein aggregation after focal brain ischemia and reperfusion. J Cereb Blood Flow Metab.

[15] Yang W, Sheng H, Warner DS, Paschen W (2008). Transient focal cerebral ischemia induces a dramatic activation of small ubiquitin-like modifier conjugation. J Cereb Blood Flow Metab.

[16] Zhang F, Liu CL, Hu BR (2006). Irreversible aggregation of protein synthesis machinery after focal brain ischemia. J Neurochem.

[17] Kim HJ (2013). Mutations in prion-like domains in hnRNPA2B1 and hnRNPA1 cause multisystem proteinopathy and ALS. Nature.

[18] Shelkovnikova TA, Robinson HK, Troakes C, Ninkina N, Buchman VL (2014). Compromised paraspeckle formation as a pathogenic factor in FUSopathies. Hum Mol Genet.

[19] Thomas-Jinu S (2017). Non-nuclear Pool of Splicing Factor SFPQ Regulates Axonal Transcripts Required for Normal Motor Development. Neuron.

[20] Stetler RA (2010). Heat shock proteins: cellular and molecular mechanisms in the central nervous system. Prog Neurobiol.

[21] DeGracia DJ, Kumar R, Owen CR, Krause GS, White BC (2002). Molecular pathways of protein synthesis inhibition during brain reperfusion: implications for neuronal survival or death. J Cereb Blood Flow Metab.

[22] Durkin JP (1997). Evidence that the early loss of membrane protein kinase C is a necessary step in the excitatory amino acid-induced death of primary cortical neurons. J Neurochem.

[23] Hara H, Onodera H, Yoshidomi M, Matsuda Y, Kogure K (1990). Staurosporine, a novel protein kinase C inhibitor, prevents postischemic neuronal damage in the gerbil and rat. J Cereb Blood Flow Metab.

[24] Vest RS, O’Leary H, Coultrap SJ, Kindy MS, Bayer KU (2010). Effective post-insult neuroprotection by a novel Ca(2+)/calmodulin-dependent protein kinase II (CaMKII) inhibitor. J Biol Chem.

[25] Waxham MN, Grotta JC, Silva AJ, Strong R, Aronowski J (1996). Ischemia-induced neuronal damage: a role for calcium/calmodulin-dependent protein kinase II. J Cereb Blood Flow Metab.

[26] Liu H (2011). Modification of ubiquitin-C-terminal hydrolase-L1 by cyclopentenone prostaglandins exacerbates hypoxic injury. Neurobiol Dis.

[27] Liu MC (2010). Ubiquitin C-terminal hydrolase-L1 as a biomarker for ischemic and traumatic brain injury in rats. Eur J Neurosci.

[28] Lai XJ (2014). Selective 14-3-3gamma induction quenches p-beta-catenin Ser37/Bax-enhanced cell death in cerebral cortical neurons during ischemia. Cell Death Dis.

[29] Wu JS (2009). Ligand-activated peroxisome proliferator-activated receptor-gamma protects against ischemic cerebral infarction and neuronal apoptosis by 14-3-3 epsilon upregulation. Circulation.

[30] Hata R, Maeda K, Hermann D, Mies G, Hossmann KA (2000). Evolution of brain infarction after transient focal cerebral ischemia in mice. J Cereb Blood Flow Metab.

[31] Rajdev S (2000). Mice overexpressing rat heat shock protein 70 are protected against cerebral infarction. Ann Neurol.

[32] Yenari MA (1998). Gene therapy with HSP72 is neuroprotective in rat models of stroke and epilepsy. Ann Neurol.

[33] Lu A, Ran R, Parmentier-Batteur S, Nee A, Sharp FR (2002). Geldanamycin induces heat shock proteins in brain and protects against focal cerebral ischemia. J Neurochem.

[34] Datwyler AL (2011). SUMO2/3 conjugation is an endogenous neuroprotective mechanism. J Cereb Blood Flow Metab.

[35] Lee YJ (2011). Elevated global SUMOylation in Ubc9 transgenic mice protects their brains against focal cerebral ischemic damage. PLoS One.

[36] Zhu Y, Bu Q, Liu X, Hu W, Wang Y (2014). Neuroprotective effect of TAT-14-3-3epsilon fusion protein against cerebral ischemia/reperfusion injury in rats. PLoS One.

[37] Yin XH, Han YL, Zhuang Y, Yan JZ, Li C (2017). Geldanamycin inhibits Fas signaling pathway and protects neurons against ischemia. Neurosci Res.

[38] Hennig S (2015). Prion-like domains in RNA binding proteins are essential for building subnuclear paraspeckles. J Cell Biol.

[39] Sama RR (2013). FUS/TLS assembles into stress granules and is a prosurvival factor during hyperosmolar stress. J Cell Physiol.

[40] Udan M, Baloh RH (2011). Implications of the prion-related Q/N domains in TDP-43 and FUS. Prion.

[41] Caldeira MV, Salazar IL, Curcio M, Canzoniero LM, Duarte CB (2014). Role of the ubiquitin-proteasome system in brain ischemia: friend or foe?. Prog Neurobiol.

[42] Ge P, Luo Y, Liu CL, Hu B (2007). Protein aggregation and proteasome dysfunction after brain ischemia. Stroke.

[43] Hu BR, Wieloch T (1993). Stress-induced inhibition of protein synthesis initiation: modulation of initiation factor 2 and guanine nucleotide exchange factor activities following transient cerebral ischemia in the rat. J Neurosci.

[44] Althausen S (2001). Changes in the phosphorylation of initiation factor eIF-2alpha, elongation factor eEF-2 and p70 S6 kinase after transient focal cerebral ischaemia in mice. J Neurochem.

[45] Mengesdorf T, Proud CG, Mies G, Paschen W (2002). Mechanisms underlying suppression of protein synthesis induced by transient focal cerebral ischemia in mouse brain. Exp Neurol.

[46] Dammer EB (2012). Coaggregation of RNA-binding proteins in a model of TDP-43 proteinopathy with selective RGG motif methylation and a role for RRM1 ubiquitination. PLoS One.

[47] Gami-Patel P, Bandopadhyay R, Brelstaff J, Revesz T, Lashley T (2016). The presence of heterogeneous nuclear ribonucleoproteins in frontotemporal lobar degeneration with FUS-positive inclusions. Neurobiol Aging.

[48] Takanashi K, Yamaguchi A (2014). Aggregation of ALS-linked FUS mutant sequesters RNA binding proteins and impairs RNA granules formation. Biochem Biophys Res Commun.

[49] Kayali F, Montie HL, Rafols JA, DeGracia DJ (2005). Prolonged translation arrest in reperfused hippocampal cornu Ammonis 1 is mediated by stress granules. Neuroscience.

[50] Jackman K, Kunz A, Iadecola C (2011). Modeling focal cerebral ischemia *in vivo*. Methods Mol Biol.

[51] Zhang S, Van Pelt CK, Henion JD (2003). Automated chip-based nanoelectrospray-mass spectrometry for rapid identification of proteins separated by two-dimensional gel electrophoresis. Electrophoresis.

[52] Yang Y (2011). Evaluation of different multidimensional LC-MS/MS pipelines for isobaric tags for relative and absolute quantitation (iTRAQ)-based proteomic analysis of potato tubers in response to cold storage. J Proteome Res.

[53] Chen JW (2013). Proteomic comparison of historic and recently emerged hypervirulent Clostridium difficile strains. J Proteome Res.

[54] Cox J, Mann M (2008). MaxQuant enables high peptide identification rates, individualized p.p.b.-range mass accuracies and proteome-wide protein quantification. Nat Biotechnol.

[55] Cox J (2014). Accurate proteome-wide label-free quantification by delayed normalization and maximal peptide ratio extraction, termed MaxLFQ. Mol Cell Proteomics.

