# Publisher Correction: Cerebral ischemia induces the aggregation of proteins linked to neurodegenerative diseases

**DOI:** 10.1038/s41598-018-25183-4

**Published:** 2018-04-26

**Authors:** Anja Kahl, Ismary Blanco, Katherine Jackman, Juhi Baskar, Harihar Milaganur Mohan, Reunet Rodney-Sandy, Sheng Zhang, Costantino Iadecola, Karin Hochrainer

**Affiliations:** 1 Feil Family Brain and Mind Research Institute, Weill Cornell Medicine, New York, NY10065 USA; 2 Institute of Biotechnology and Life Sciences Biotechnologies, Cornell University, Ithaca, NY14853 USA

Correction to: *Scientific Reports* 10.1038/s41598-018-21063-z, published online 09 February 2018

The original PDF version of this Article contained a truncated Figure 1. This error has now been corrected in the PDF version of the Article; the HTML version was correct from the time of publication.

