# Supplementary material for: Cerebral ischemia induces the aggregation of proteins linked to neurodegenerative diseases
Source: Sci Rep. 2018 Feb 9;8:2701. doi: 10.1038/s41598-018-21063-z (PMC5807442; doi:10.1038/s41598-018-21063-z)

Cerebral ischemia induces the aggregation of proteins linked to neurodegenerative diseases

Anja Kahl[1], Ismary Blanco[1], Katherine Jackman, Juhi Baskar, Harihar Milaganur Mohan, Reunet Rodney-Sandy, Sheng Zhang[#], Costantino Iadecola and Karin Hochrainer[*]

Feil Family Brain and Mind Research Institute, Weill Cornell Medicine, New York, NY10065, USA

[#]Institute of Biotechnology and Life Sciences Biotechnologies, Cornell University, Ithaca, NY14853, USA

[1]These authors contributed equally to this work

*Corresponding Author:

407 East 61st Street, RR-406, New York, NY10065, USA; Phone 1-646-962-8258; Fax 1-646-962-0535; Email kah2015@med.cornell.edu.

Supplementary Figure S1: high-resolution image to Figure 1E

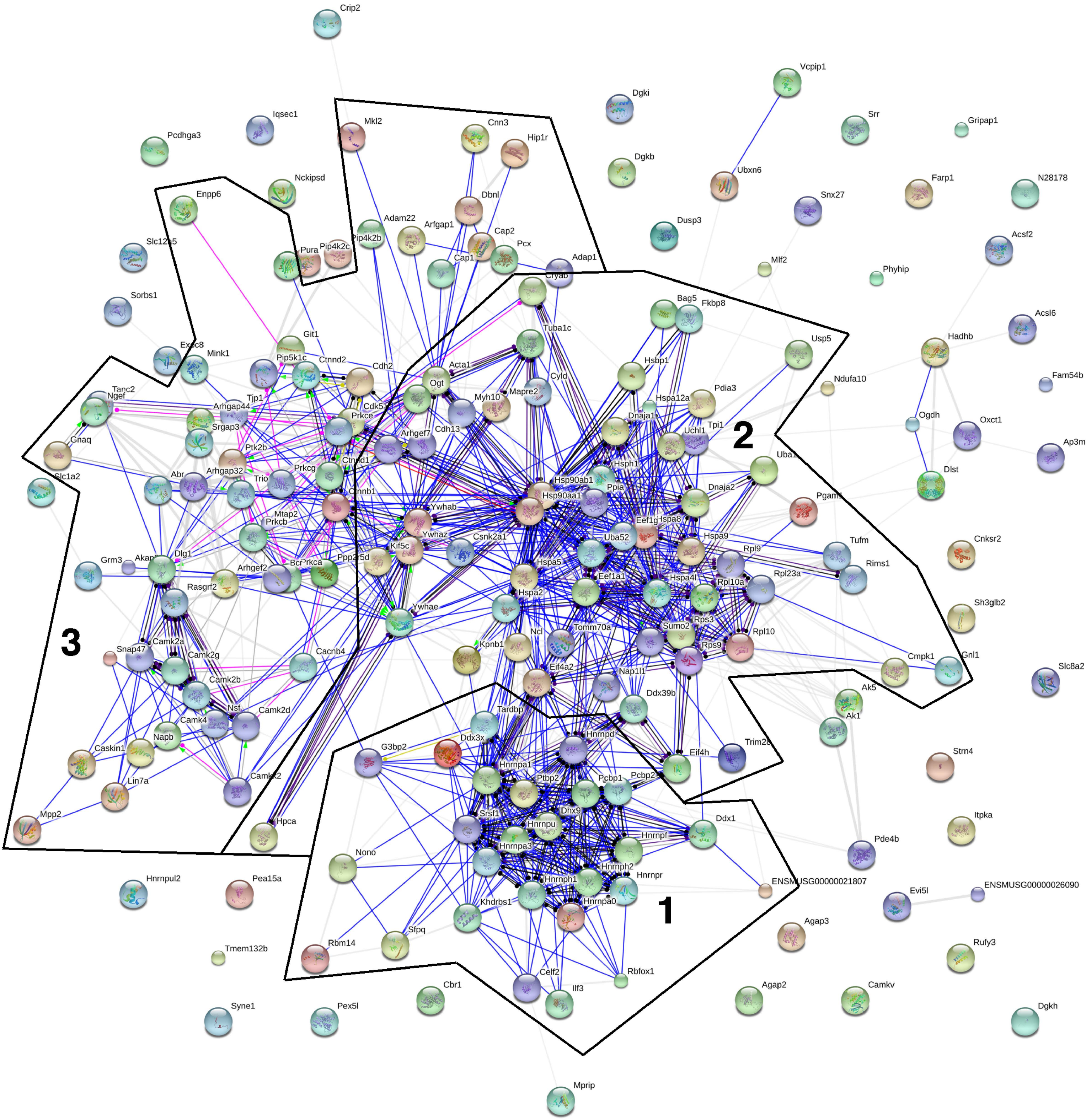

Supplementary Figure S2: full-size blots to Figure 2B

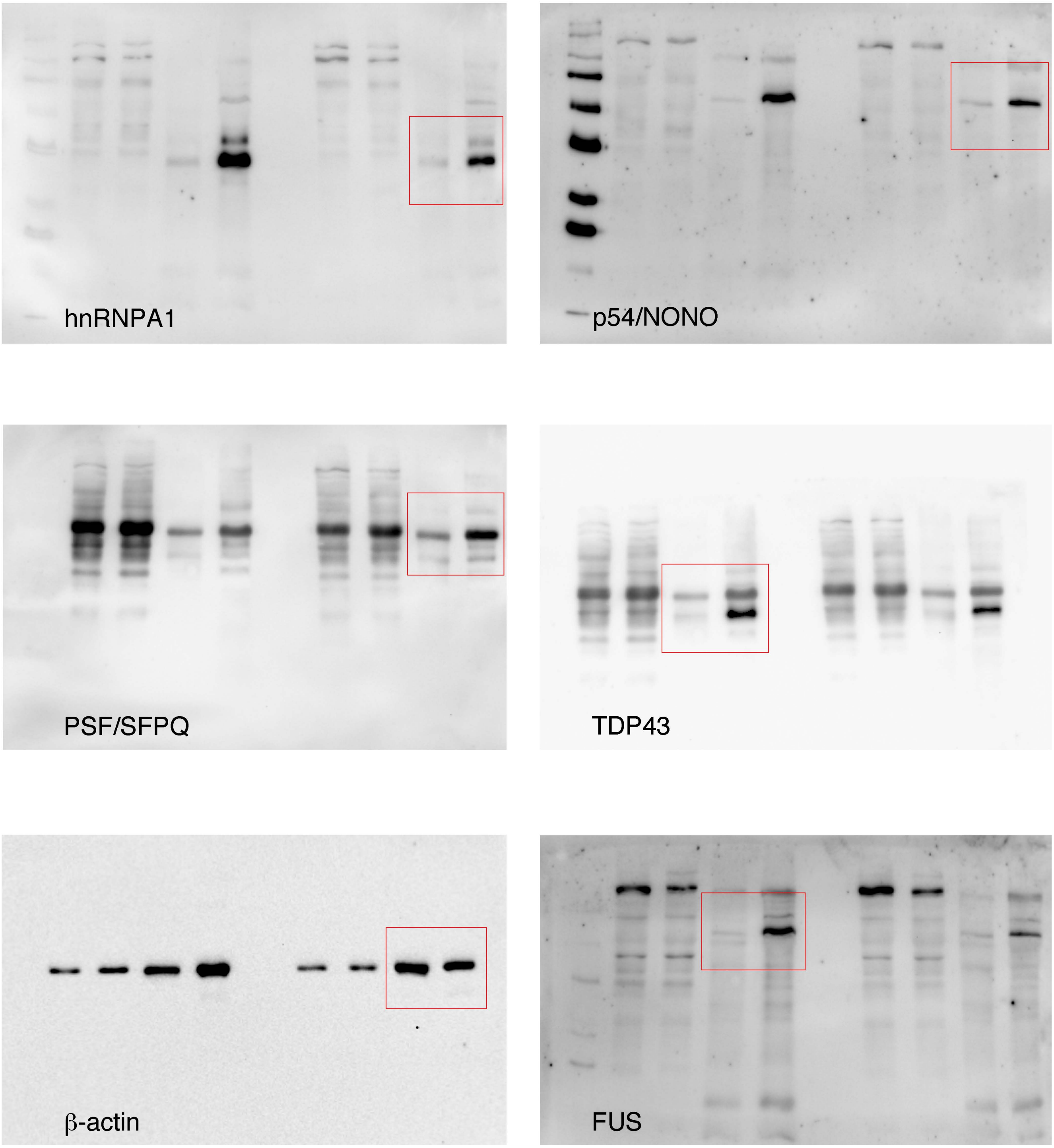

Supplementary Figure S3: full-size blots to Figure 2C

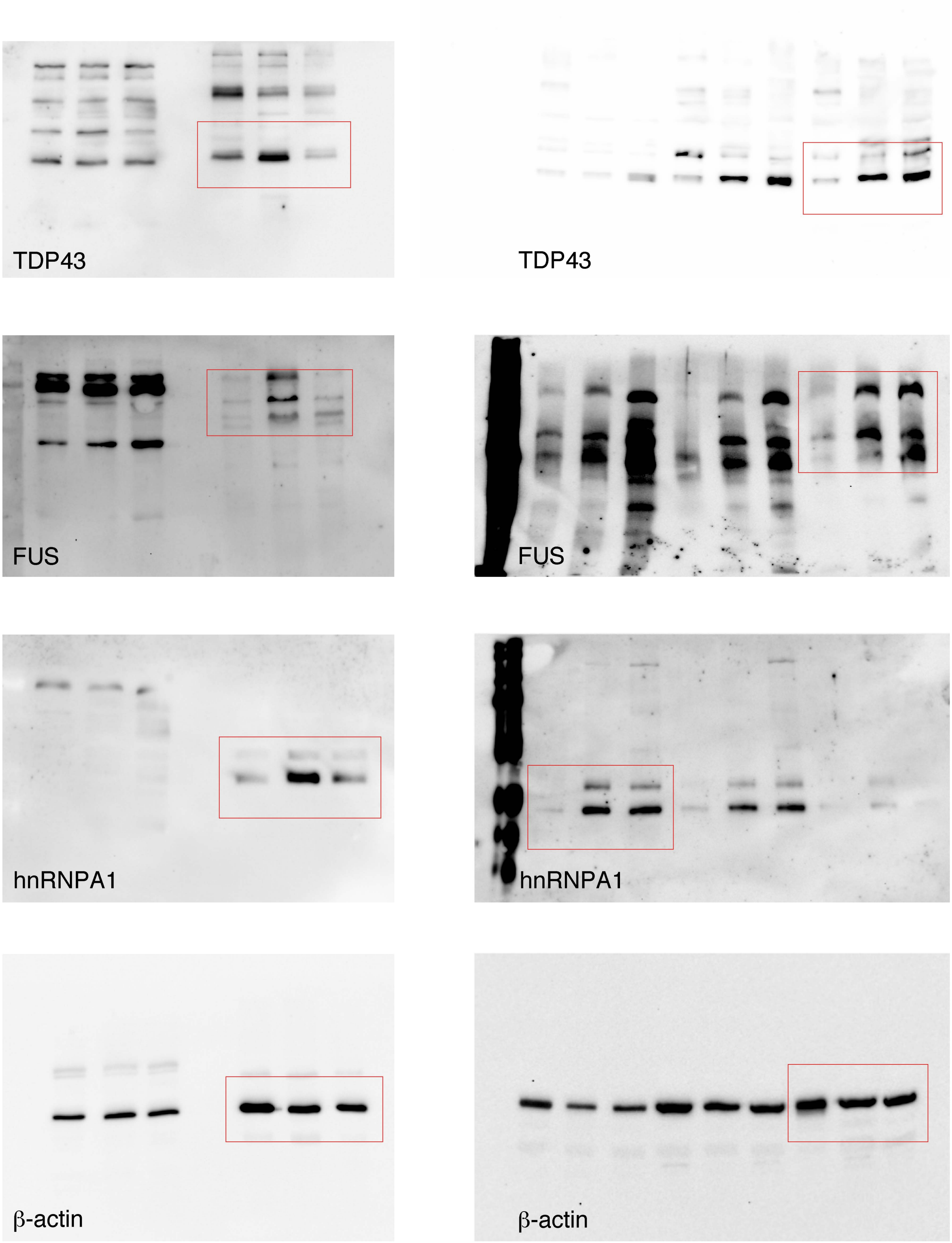

Supplement: Supplementary file 1 — Supplementary Figures S1-S3 [file 41598_2018_21063_MOESM1_ESM.pdf]
